# Supplementary figures and images for: Opening the digital doorway to sexual healthcare: Recommendations from a behaviour change wheel analysis of barriers and facilitators to seeking online sexual health information and support among underserved populations
Source: PLoS One. 2025 Jan 8;20(1):e0315049. doi: 10.1371/journal.pone.0315049 (PMC11709294; doi:10.1371/journal.pone.0315049)

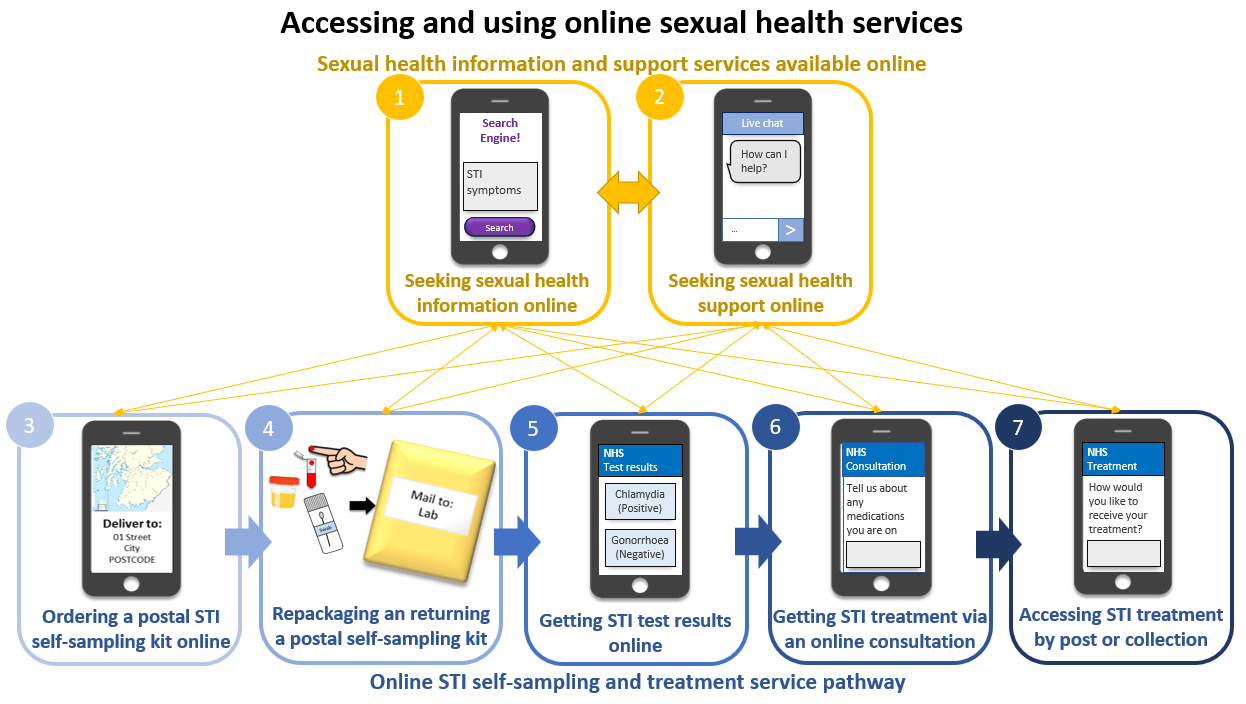

Supplement: S1 Fig — Thick yellow arrows indicate a non-sequential order, where the elements of care can occur in any order, e.g., getting sexual health information online can occur before or getting sexual health support online. Thick blue arrows indicate a sequential order, where a later element of care cannot precede an earlier domain, e.g., getting STI test result online must occur after getting a postal STI/BBV self-sampling kit online. Thin yellow arrows indicate that getting sexual health information or support online can occur at any point in the STI self-sampling and treatment pathway. (TIF) [file pone.0315049.s001.tif]

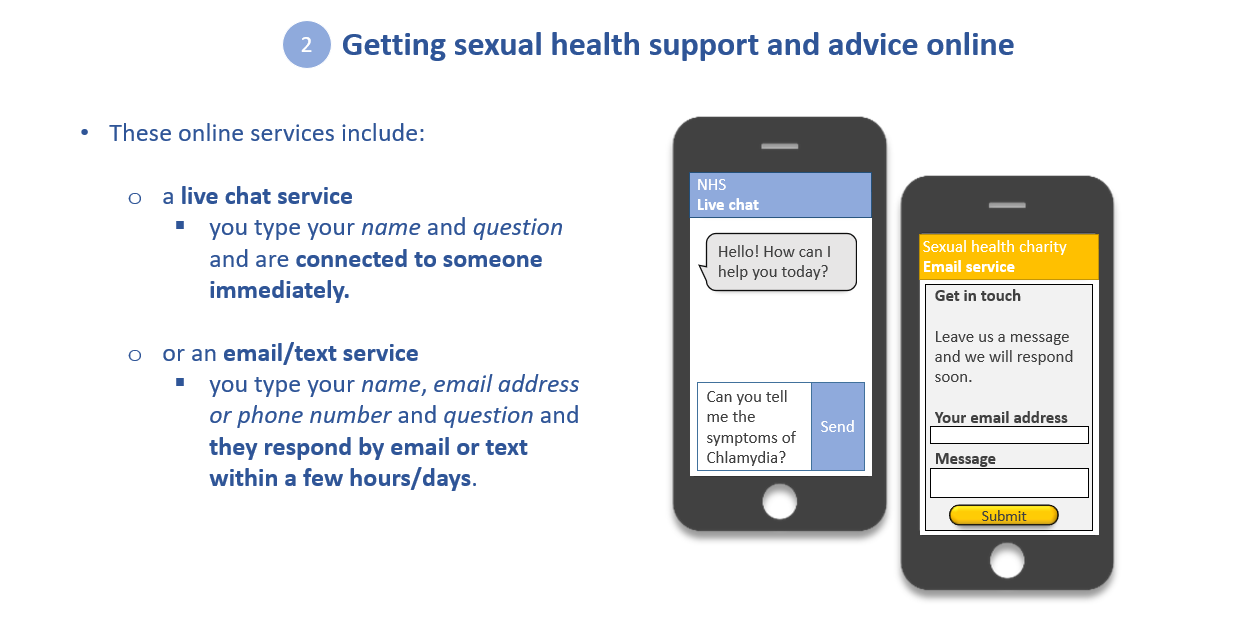

Supplement: S2 Fig — (TIF) [file pone.0315049.s002.tif]
