# Supplementary material for: Opening the digital doorway to sexual healthcare: Recommendations from a behaviour change wheel analysis of barriers and facilitators to seeking online sexual health information and support among underserved populations
Source: PLoS One. 2025 Jan 8;20(1):e0315049. doi: 10.1371/journal.pone.0315049 (PMC11709294; doi:10.1371/journal.pone.0315049)
Supplement: S1 Table — (DOCX) [file pone.0315049.s003.docx]

| **Online sexual health information/support** | **Website name** | **Website link** |
| --- | --- | --- |
| **Online sexual health information** | | |
| Information on websites | NHS | <https://www.nhs.uk/service-search/sexual-health> |
|  | Brook | <https://www.brook.org.uk/> |
|  | SH:24 | <https://sh24.org.uk/> |
|  | Terrance Higgins Trust | <https://www.tht.org.uk/> |
|  | Waverly care | <https://www.waverleycare.org/> |
| **Online sexual health support** | | |
| Synchronous communication (live chat) with trained professional | Terrance Higgins Trust | <https://www.tht.org.uk/our-services/support-services/tht-direct-helpline> |
| Synchronous communication (live chat) with automated responses (chatbot) | Planned Parenthood | <https://roo.plannedparenthood.org/> |
|  | PositiveEast | <https://www.positiveeast.org.uk/chattopat/> |
|  | Brook | <https://www.brook.org.uk/contact-us/> |
|  | NHS Blackpool Teaching Hospitals | <https://www.lancashiresexualhealth.nhs.uk/sexual-health-guides/chatbot> |
| Asynchronous communication (email) with a trained professional | Embrace | <https://www.embracewolverhampton.nhs.uk/about-us/chat-sexual-health#:~:text=Text%20a%20sexual%20health%20nurse,Smear%20tests> |
|  | Sandyford | <https://www.sandyford.scot/about-us/contact-us/#:~:text=If%20you%20are%20involved%20in,.scot.nhs.uk>. |
|  | Umbrella | <https://umbrellahealth.co.uk/our-services/chathealth/> |
|  | Sexual Health Heartfordshire | <https://www.sexualhealthhertfordshire.clch.nhs.uk/support/chat-sexual-health> |
|  | Waverly care | <https://www.waverleycare.org/home/contact-us/> |
|  | Terrance Higgins Trust | <https://www.tht.org.uk/get-help/support-services/tht-direct-helpline> |
|  | SH:24 | <https://sh24.org.uk/contact-us> |
| Asynchronous communication (SMS text) with a trained professional | Leeds Sexual Health | <https://www.leedssexualhealth.com/wheretogo/chat-health> |
|  | NHS Dorset HealthCare University | https://sexualhealthdorset.org/how-can-we-help/chat-sexual-health/ |
