# Supplementary material for: Opening the digital doorway to sexual healthcare: Recommendations from a behaviour change wheel analysis of barriers and facilitators to seeking online sexual health information and support among underserved populations
Source: PLoS One. 2025 Jan 8;20(1):e0315049. doi: 10.1371/journal.pone.0315049 (PMC11709294; doi:10.1371/journal.pone.0315049)
Supplement: S2 Table — aSES = Socio-economic Status. bIMD = Index of Multiple Deprivation [70]. cBold = Target met or exceeded. (DOCX) [file pone.0315049.s004.docx]

| **Demographic characteristics and socioeconomic indicators** | | **Targets** | **Final sample** ^c^ |
| --- | --- | --- | --- |
| **PROGRESS** | | | |
| **Place of residence**  **(SES** ^a^**)** | **Area deprivation (IMD** ^b^) | n=10-15 from the most deprived quintiles | **n=17** from the most deprived quintiles  (n=9 most deprived, n=8 second most deprived) |
| **Race/Ethnicity** | **Ethnicity** | n=10 from minoritised ethnic groups  (especially Black Caribbean, Black Other, and Mixed ethnic populations) | **n=9** from minoritised ethnic groups  (n=3 Black African, n=1 Mixed/other (Black African and White), n=4 Asian Pakistani, n=1 Asian Chinese) |
| **Occupation (SES** ^a^**)** | **Employment** | n=10 out of work / in temporary work | **n=11** out of work |
| **Gender/Sex** | **Gender** | n=10-15 cisgender men | **n=16** cisgender men |
|  |  | n=10-15 cisgender women | **n=16** cisgender women |
|  |  | n=5-10 non-binary and trans | n=2 non-binary and trans  (n=1 non-binary, n=1 non-binary/transmasc) |
| **Education (SES** ^a^**)** | **Education** | n=10-15 with no higher education | **n=18** no higher education  (n=6 high school, n=12 college) |
| **Plus** | | | |
| **Age** | **Age** | n=15 aged under 25 years | n=8 aged under 25 years |
|  |  | n=10 aged 25 to 49 years | **n=23** aged 25-49 |
|  |  | n=5 aged 50+ years | n= 3 aged 50+ |
| **Sexual orientation/ behaviour/ identity** | **Sexuality** | n=5-10 gay and bisexual men who have sex with men (GBMSM) | **n=9** GBMSM  (n=2 bisexual, n=7 gay) |
|  |  | n=15-20 heterosexual men | n=6 heterosexual men |
|  |  | n=5 heterosexually identifying men who have sex with men (HSMSM) | n=0 HIMSM |
|  |  | n=5 bisexual women | **n=6** bisexual women |
| **Disability** | **Disability** | n=5 with a mild learning disability | **n=10** with a learning disability |
