# Supplementary material for: Opening the digital doorway to sexual healthcare: Recommendations from a behaviour change wheel analysis of barriers and facilitators to seeking online sexual health information and support among underserved populations
Source: PLoS One. 2025 Jan 8;20(1):e0315049. doi: 10.1371/journal.pone.0315049 (PMC11709294; doi:10.1371/journal.pone.0315049)
Supplement: S3 Table — (DOCX) [file pone.0315049.s005.docx]

| **Eligibility Questions** |
| --- |
| 1. Are you aged 16 or over? |
| 1. Do you live in the UK? |
| 1. Do you have you have access to the internet? |
| 1. Have you had sex in the last 12 months? |
| 1. a) Have you ever ordered a postal STI self-sampling kit before?   b) *[If yes]* Did you experience any difficulty in ordering it? |
| **PROGRESS+ screening questions:** |
| 1. What age are you? |
| 1. How would you best describe your gender? |
| 1. Were you assigned a different gender at birth? |
| 1. How would you describe your sexual orientation? |
| 1. How would you describe your ethnicity? |
| 1. What is your highest educational qualification? |
| 1. How would you describe your current occupation? |
| 1. What is your postcode? |
| 1. a) Do you have any physical or mental health conditions or illnesses that have lasted or are expected to last for 12 months or more?   b) *[If yes]* Do any of your illnesses or conditions reduce your ability to carry out day to day activities? |
| 1. Do you consider yourself to have a learning disability? |
| **PROGRESS+ demographic questions:** |
| 1. a) Were you born in the UK?   b) *[If no]* Which country were you born in? |
| 1. What is your first language? |
| 1. a) Do you regard yourself as belonging to any particular religion or faith?   b) *[If yes]* Which religion or faith? |
| 1. Do you ever have difficulty making ends meet at the end of the month? |
| 1. How many people can you call upon to provide support if you need it? |
| **Internet access and use questions:** |
| 1. How do you typically access the Internet? |
| 1. What device(s) do you use to access the internet? |
| 1. Are these your own devices? |
| 1. How often do you go online? |
| 1. What sorts of things do you go online for? |
| 1. a) Have you ever searched for ~~health or~~ sexual health information online?   b) *[if yes]* What sort of information have you searched for? |
| 1. a) Have you ever used any online ~~health or~~ sexual health services?   b) *[if yes]* Which services have you used?   - prompt about live chat/email or text service |
| 1. How would you rate your skills in using the internet? |
