# Supplementary material for: Opening the digital doorway to sexual healthcare: Recommendations from a behaviour change wheel analysis of barriers and facilitators to seeking online sexual health information and support among underserved populations
Source: PLoS One. 2025 Jan 8;20(1):e0315049. doi: 10.1371/journal.pone.0315049 (PMC11709294; doi:10.1371/journal.pone.0315049)
Supplement: S4 Table — (DOCX) [file pone.0315049.s006.docx]

| **Behavioural domain**  Description of element of online sexual healthcare and corresponding slide | **Specific behaviour** |  | **Question** | **Prompts** |
| --- | --- | --- | --- | --- |
| **Knowledge of current online sexual healthcare (1 min)** | **-** |  | *[If never searched for/used SHS online before]*  **Can you briefly tell me about your experience of accessing sexual health services in the past?** | - How did you become aware of these health / sexual health services? - What, if anything, has affected your decision not to use online sexual health services? - What do you think about online sexual health services as an idea?   - What do you think it may be like?   - What do you think it might involve? - Do you have a good idea of what STIs are? |
|  |  |  | *[If searched for/used SHS online before]*  **Can you briefly tell me, from your recent experience, what you know about online sexual health services/ health services?** | - How did you become aware of these online sexual health services? - Can you tell me what has affected your decision to use online sexual health services? |
| **Online sexual healthcare *per se* (2 mins)** | **Access online sexual healthcare** |  | **Is there anything that might make/has made it difficult for you to access online sexual health services?** | - Is there anything that might make it difficult for you to get online to use online SHS? - Think about how you normally access the internet |
|  | **Use online sexual healthcare** |  | **Is there anything that might/has make it difficult for you to use online sexual health services?** |  |
| **1. Getting sexual health information online (5-7 mins)**  *[#1 on slide 3 & Slide 4]*  You can find a wide range of information about sexual health online, on websites like the NHS and sexual health charity websites. These websites have information on them about sexual health services, including clinic locations and opening times. They also have information about sexual health, including how to have safe sex and STI symptoms and testing  *[Slide 5]*  You can search for this information on Internet, using devices like a mobile phone or computer.  *[Sense check]* | **-** | Previous experience | *[If never searched for sexual health or health information online before]*  **Tell me about your experience using the internet for any kind of information** | - What, if anything, has stopped you from searching for health/sexual health information online? - Compared to getting sexual health information in person or on the phone |
|  |  |  | *[If only searched for other health information]*  **Tell me about any experience of searching for other health information online** | - What are your thoughts on searching for SH information online? |
|  |  |  | *[If have searched for sexual health information online before]*  **Tell me about your previous experience of searching for sexual health information online** | - What are your thoughts on searching for SH information online? |
|  |  | Barriers | **What might make/ has made it difficult for you to search for sexual health information online?** | - Why? - What might you struggle with? - Is there anything that might stop you/put you off? - Is there anything you might be concerned about? - How would you know if you could trust the website? - What challenges may there be for you in searching for sexual health information online? |
|  |  | Facilitators | **What might make/has made it easy for you to search for sexual health information online?** | - Why? - What are the benefits for you to this? - What might encourage you to do this? - What would help you do this? - What do you think would be good about it? Why? |
| **2. Getting sexual health information and advice online (5-7 mins)**  *[#2 on slide 3 & slide 6]*  Some sexual health clinic and charity websites offer a service that lets you talk to a sexual healthcare professional (doctor or nurse on the NHS) or trained staff (on a sexual health charity website). You can ask or talk about things like safe sex, sexually transmitted infections, and the services available to you.  These are screenshots of these services from a sexual health website.  *[Slide 7]*  This includes a live chat service where you type your name and a question and are connected to someone immediately, or an email or text service here you type your name, email address or phone number and a question, and they respond to you by email or text as soon as possible (generally within a few hours to days)  *[Sense check]* | **-** |  | *[If never got sexual health support and advice online before]*  **What do you think about getting sexual health support and advice online?** | - What might be different for you about using a live chat and an email or text service to get sexual health support and advice online? - Do you think there might be any difference between talking to a HCP or a trained member of staff |
|  | **Live chat** |  | *[If never used a live chat for getting sexual health support and advice online before]*  **What do you think about using a live chat service to get sexual health support and advice online?** | - Why? |
|  |  | Barriers | **What would make/ has made it difficult for you to use a live web chat to get sexual health support and advice online?** | - Is there anything you might be concerned about? - Is there anything that would stop you? - What might put you off using a live web chat for getting sexual health support and advice? - Why wouldn’t you do this? - What challenges might there be for you? - What might help you with this? |
|  |  | Facilitators | **What would make/ has made it easy for you to use a live web chat to get sexual health support and advice online?** | - What do you think would encourage you to use a live web chat for getting sexual health support and advice? - Why would you do this? - Difference between talking to HCP and trained staff? |
|  | **Email/Text service** |  | *[If never used an email/text service for getting sexual health support and advice online before]*  **What do you think about using an email/text service to get sexual health support and advice online?** | - Why? |
|  |  | Barriers | **What would make/ has made it difficult for you to use an email/text service to get sexual health support and advice online?** | - What might stop you? - What might put you off using an email/text service for getting sexual health support and advice? - Why wouldn’t you do this? - What challenges might there be? - What might help you with this? |
|  |  | Facilitators | **What would make/ has made it easy for you to use an email or text service to get sexual health support and advice online?** | - What would encourage you to use an email/text service to get sexual health support and advice? - Why would you do this? |
|  | **Sexual health service** | Barriers |  | - Is there anything that would make it difficult for you to collect your STI treatment from a pharmacy? - Challenges for you |
|  |  | Facilitators |  | - Is there anything that would make it easy for you to collect your STI treatment from a SHS? - Benefits for you |
