# Supplementary material for: Opening the digital doorway to sexual healthcare: Recommendations from a behaviour change wheel analysis of barriers and facilitators to seeking online sexual health information and support among underserved populations
Source: PLoS One. 2025 Jan 8;20(1):e0315049. doi: 10.1371/journal.pone.0315049 (PMC11709294; doi:10.1371/journal.pone.0315049)
Supplement: S5 Table — (DOCX) [file pone.0315049.s007.docx]

| **Sources of support**  We want to make sure that you have a list of services that offer different types of support and information in case something came up in the interview that was difficult or uncomfortable for you. | |
| --- | --- |
| **Sexual health services and organisations** | |
| **Name and details of support offered** | **Website and contact details** |
| *Sandyford Sexual Health Service* offers sexual healthcare for those living in the Glasgow area | <https://sandyford.scot> \| 0141 211 8130 \| [sandyford@ggc.scot.nhs.uk](mailto:sandyford@ggc.scot.nhs.uk) |
| *The Chalmers Centre* offers sexual healthcare for those living in the Lothian area | <https://lothiansexualhealth.scot> \| 0131 536 1070 |
| To find your nearest sexual health service visit: | <https://sexualhealthscotland.co.uk/get-help/sexual-health-service-finder> |
| *S-X* provide information on sexual health, relationships, and mental health for gay and bisexual men, and all men who have sex with men living in Scotland (including testing services) | <https://s-x.scot> \| 0131 652 3250 or 07703 840970 \| [info@s-x.scot](mailto:info@s-x.scot) |
| *Terrence Higgins Trust* provide information on sexual health, safer sex, and sexually transmitted infections, including testing services | <https://tht.org.uk/sexual-health> \| 0808 802 1221 (direct helpline) |
| *Waverley Care* provide information on sexual health, sex, and relationships | <https://www.waverleycare.org/support-and-advice/sexual-health> |
| *The African Health Project* provide support and advice on HIV and sexual health (and a range of other health and social care issues) | 0141 332 2520 \| 0131 558 1425 |
| *Young Scot* offer information about sex, sexual health, and relationships for young people living in Scotland | <https://young.scot/campaigns/national/sexual-health> |
|  | |
| **Wider health services and organisations** | |
| **Name and details of support offered** | **Website and contact details** |
| *NHS Inform* provide up-to-date information about COVID-19 | <https://www.nhsinform.scot/illnesses-and-conditions/infections-and-poisoning/coronavirus-covid-19> |
| *NHS 24* provide urgent care advice and mental health support | <https://www.nhs24.scot> \| 111 |
| *Breathing Space* have experienced advisors who will listen and offer information and advice regarding mental health | <https://breathingspace.scot> \| 0800 83 85 87 |
| *Hwupenyu Health and Wellbeing Project* offer information, support, and guidance on health, mental health, and social topics for Black ethnic communities living in Scotland | <http://www.hwupenyuproject.org/> \| 0141 418 0940 \| [info@hwupenyuproject.org](mailto:info@hwupenyuproject.org) |
| *The Scottish Refugee Council* provide practical support, advice, and a listening ear for people in need of refugee protection | <https://www.scottishrefugeecouncil.org.uk/contact-us/> \| 0808 196 7274 |
| *Switchboard* provide an information, support and referral service for LGBTQ+ people | <https://switchboard.lgbt/> \| 0300 330 0630 \| [chris@switchboard.lgbt](mailto:chris@switchboard.lgbt) |
| *Disability Information Scotland* provide reliable, accurate, and accessible information for people living with disability in Scotland | <https://www.disabilityscot.org.uk/> \| 0300 323 9961 |
| *Victim Support Scotland* provide support and information to people affected by crime, including but not limited to domestic abuse and sexual assault | <https://victimsupport.scot> \| 0800 160 1985 |
