# Supplementary material for: Opening the digital doorway to sexual healthcare: Recommendations from a behaviour change wheel analysis of barriers and facilitators to seeking online sexual health information and support among underserved populations
Source: PLoS One. 2025 Jan 8;20(1):e0315049. doi: 10.1371/journal.pone.0315049 (PMC11709294; doi:10.1371/journal.pone.0315049)
Supplement: S6 Table — aParticipant demographics for one participant were not obtained, table includes demographics for n = 34, except where participants did not wish to answer the question. Percentages are calculated for N = 35. (DOCX) [file pone.0315049.s008.docx]

| **Variables** | **n** | **%** ^a^ |
| --- | --- | --- |
| **Digital literacy** | | |
| **Skills using the internet** |  |  |
| High (8-10; pretty good, confident, excellent) | 20 | 57.1 |
| Medium (4-7; not great, getting better, not bad) | 12 | 34.3 |
| Low (0-3) | 2 | 5.7 |
| **Experience using the internet for sexual health** | | |
| **Previously searched for health or sexual health information** |  |  |
| Yes | 20 | 57.1 |
| No | 11 |  |
| Yes, health only, not sexual health | 2 | 5.7 |
| Yes, tried and struggled | 1 | 2.9 |
| **Sexual health information searched for** |  |  |
| Sexually transmitted infections (STIs)/ blood borne viruses (BBV) symptoms (including yeast infection, thrush, urinary tract infections) | 11 | 31.4 |
| Information about STIs/BBV (including most common, how they’re contracted) | 7 | 20.0 |
| How/where to get tested | 5 | 14.3 |
| Where to find local clinics | 3 | 8.6 |
| Information about contraception (including the coil, morning after pill) | 3 | 8.6 |
| Information about HIV (including support groups, pre-exposure prophylaxis) | 2 | 5.7 |
| Treatment for STIs/HIV (e.g., what the options are, where to get treatment) | 2 | 5.7 |
| Window periods | 2 | 5.7 |
| Efficacy and reliability of tests | 1 | 2.9 |
| Trans specific sexual health | 1 | 2.9 |
| Sexual health in general | 1 | 2.9 |
| **Previous use of online sexual health services** |  |  |
| Live chat or email/text exchange service | 0 | 0 |
| Booking appointment for in-person clinic online | 4 | 11.4 |
| Ordering medication (private clinic) | 1 | 2.9 |
| **Ordered or used an online postal STI/BBV self-sampling (OPSS) kit** |  |  |
| Never ordered an OPSS | 24 | 68.6 |
| Ordered and struggled to use an OPSS (e.g., blood sampling) | 7 | 20.0 |
| Struggled to order an OPSS | 3 | 8.6 |
| **Experience using the internet** | | |
| **Devices used to access the internet** |  |  |
| Laptop/computer and/or mobile phone | 20 | 57.1 |
| Phone only | 6 | 17.1 |
| Multiple devices (e.g., smart TV, mobile phone, tablet, computer, laptop) | 4 | 11.4 |
| Phone and tablet | 2 | 5.7 |
| Tablet only | 1 | 2.9 |
| iPad | 1 | 2.9 |
| WiFi | 1 | 2.9 |
| **Own device owned** |  |  |
| Yes | 30 | 85.7 |
| Yes, phone only | 3 | 8.6 |
| No | 1 | 2.9 |
| **How often online** |  |  |
| Every day/daily | 25 | 71.4 |
| Constantly/all the time/every hour | 4 | 11.4 |
| Weekly or monthly | 2 | 5.7 |
| Not often/not too much | 2 | 5.7 |
| Once or twice a week | 1 | 2.9 |
| **What participants go online for** |  |  |
| Social media (e.g., Facebook, Instagram, Twitter, WhatsApp) | 22 | 62.9 |
| School/University work (e.g., homework, research, classwork/coursework) | 12 | 34.3 |
| TV streaming (e.g., YouTube, Netflix) | 10 | 28.6 |
| Engine searches (e.g., how to spell words, “NHS searches”, google images) | 9 | 25.7 |
| Online shopping | 7 | 20.0 |
| News | 6 | 17.1 |
| Work (e.g., rotas, research) | 5 | 14.3 |
| Checking emails | 5 | 14.3 |
| Online groups (e.g., prayer group, Zoom meetings) | 3 | 8.6 |
| Contacting friends/family | 3 | 8.6 |
| Online gaming | 2 | 5.7 |
| Steaming music | 2 | 5.7 |
| Entertainment | 1 | 2.9 |
| Booking appointments | 1 | 2.9 |
| Online banking | 1 | 2.9 |
| Meditation/motivational speeches | 1 | 2.9 |
| “Random” | 1 | 2.9 |
