# Supplementary material for: Opening the digital doorway to sexual healthcare: Recommendations from a behaviour change wheel analysis of barriers and facilitators to seeking online sexual health information and support among underserved populations
Source: PLoS One. 2025 Jan 8;20(1):e0315049. doi: 10.1371/journal.pone.0315049 (PMC11709294; doi:10.1371/journal.pone.0315049)
Supplement: S7 Table — Adapted from Michie et al. [45]. (DOCX) [file pone.0315049.s009.docx]

| **Intervention Function** | **Definition of Intervention Function** |
| --- | --- |
| Education | Increasing knowledge or understanding |
| Persuasion | Using communication or induce positive or negative feelings or stimulate action |
| Training | Imparting skills |
| Modelling | Providing an example for people to aspire to or imitate |
| Environmental restructuring | Changing the physical or social context |
| Enablement | Increasing means/reducing barriers to increase capability (beyond education and training) and opportunity (beyond environmental restructuring) |
