# Supplementary material for: Opening the digital doorway to sexual healthcare: Recommendations from a behaviour change wheel analysis of barriers and facilitators to seeking online sexual health information and support among underserved populations
Source: PLoS One. 2025 Jan 8;20(1):e0315049. doi: 10.1371/journal.pone.0315049 (PMC11709294; doi:10.1371/journal.pone.0315049)
Supplement: Attachment — Submitted filename: Response to Reviewers.docx [file pone.0315049.s010.docx]

Dear Editor,

We thank you and the reviewers for your quick and insightful review of our paper and the helpful feedback provided.

We have addressed each of your and the reviewers’ comments, detailed in four tables below, and believe the paper has benefitted greatly from this.

We look forward to your further review of this paper.

Kind regards,

Julie McLeod

**1) Journal Requirements**

**When submitting your revision, we need you to address these additional requirements.**

| **Changes requested** | **Changes made** |
| --- | --- |
| **1. Please ensure that your manuscript meets PLOS ONE's style requirements, including those for file naming. The PLOS ONE style templates can be found at:**   - <https://journals.plos.org/plosone/s/file?id=wjVg/PLOSOne_formatting_sample_main_body.pdf> - <https://journals.plos.org/plosone/s/file?id=ba62/PLOSOne_formatting_sample_title_authors_affiliations.pdf> | The title page has been updated to match the link:   - Title changed to point 12 and moved down to line 4 - Initials in brackets added after corresponding author email   The manuscript has been updated to match the link:   - All level 1 headings now bold - All paragraphs indented - New lines removed between headings and paragraphs - Space (6pt) added after all new lines - Table 1 formatting updated   File names and supplementary file names have all been updated within the manuscript and actual files for submitting |
| **2. We note that the grant information you provided in the ‘Funding Information’ and ‘Financial Disclosure’ sections do not match.** When you resubmit, please ensure that you provide the correct grant numbers for the awards you received for your study in the ‘Funding Information’ section.  **3.** Thank you for stating the following financial disclosure:  This research is part of the SEQUENCE Digital programme funded by the National Institute for Health Research (<https://www.sequencedigital.org.uk/about>).  Please state what role the funders took in the study.  If the funders had no role, please state: **"The funders had no role in study design, data collection and analysis, decision to publish, or preparation of the manuscript."** If this statement is not correct you must amend it as needed. Please include this amended Role of Funder statement in your cover letter; we will change the online submission form on your behalf. | Thank you for raising this issue. The grant information provided in the ‘Funding Information’ is correct. The Financial Disclosure Statement has been updated in the cover letter with the information requested and provided (below):  This study/project is funded by the National Institute for Health and Care research (NIHR) Programme Grants for Applied Research, NIHR2000856 (<https://fundingawards.nihr.ac.uk/award/NIHR200856>) (awarded to author C. S. E.). The views expressed are those of the author(s) and not necessarily those of the NIHR or the Department of Health and Social Care. The funders had no role in study design, data collection and analysis, decision to publish, or preparation of the manuscript. No authors received salaries or other funding from commercial companies. Central and North West London (CNWL) NHS Foundation Trust (<https://www.cnwl.nhs.uk/>) is the project Sponsor. |
| **4.** In this instance it seems there may be acceptable restrictions in place that prevent the public sharing of your minimal data. However, in line with our goal of ensuring long-term data availability to all interested researchers, PLOS’ Data Policy states that **authors cannot be the sole named individuals responsible for ensuring data access** (<http://journals.plos.org/plosone/s/data-availability#loc-acceptable-data-sharing-methods>). Data requests to a non-author institutional point of contact, such as a data access or ethics committee, helps guarantee long term stability and availability of data. Providing interested researchers with a durable point of contact ensures data will be accessible even if an author changes email addresses, institutions, or becomes unavailable to answer requests.  Before we proceed with your manuscript, please also provide non-author contact information (phone/email/hyperlink) for a data access committee, ethics committee, or other institutional body to which data requests may be sent. If no institutional body is available to respond to requests for your minimal data, please consider if there any institutional representatives who did not collaborate in the study, and are not listed as authors on the manuscript, who would be able to hold the data and respond to external requests for data access? If so, please provide their contact information (i.e., email address). Please also provide details on how you will ensure persistent or long-term data storage and availability.  **5. We note that this data set consists of interview transcripts. Can you please confirm that all participants gave consent for interview transcript to be published?**  **If the participants DID NOT provide consent for these transcripts to be published:**  - Provide a de-identified version of the data or excerpts of interview responses  - Provide information regarding how these transcripts can be accessed by researchers who meet the criteria for access to confidential data, including:  a) the grounds for restriction  b) the name of the ethics committee, Institutional Review Board, or third-party organization that is imposing sharing restrictions on the data  c) a non-author, institutional point of contact that is able to field data access queries, in the interest of maintaining long-term data accessibility.  d) Any relevant data set names, URLs, DOIs, etc. that an independent researcher would need in order to request your minimal data set.  For further information on sharing data that contains sensitive participant information, please see:   - <https://journals.plos.org/plosone/s/data-availability#loc-human-research-participant-data-and-other-sensitive-data> | Thank you for this. We appreciate that PLOS ONE is a journal of open science and we aspire to meet this. However, on this occasion, due to the restrictions of the consent obtained at the time of ethical approval and data collection, there are limitations on sharing of the interview data for this work. We understand that a non-author contact is required to ensure future access to the data and have updated the submission to include this. The submission form has been updated to include the following:  Due to the sensitive nature of the questions asked in this study, participants were assured that transcripts would remain private and confidential and would not be shared beyond the use of anonymised illustrative quotes in publications about the research. In line with our ethical approvals form Glasgow Caledonian University (GCU) Nursing and Community Health Research Ethics Committee (HLS/NCH/20/045) and the East of England - Cambridge South Research Ethics Committee (21/EE/0148), participants were not asked and did not consent to sharing their full transcripts.  The transcripts will be stored long-term, for a minimum of 10 years (from 2021) on a secure GCU network drive. Data access requests may be made to the corresponding author, Julie McLeod (julie.mcleod@gcu.ac.uk), the SEQUENCE digital team (cnwl.sequencedigital@nhs.net) or the Glasgow Caledonian University Research Ethics Committee ([hlsethics@gcu.ac.uk](mailto:hlsethics@gcu.ac.uk)) and the East of England - Cambridge South Research Ethics Committee ([cambridgesouth.rec@hra.nhs.uk](mailto:cambridgesouth.rec@hra.nhs.uk)). |
| **6. Please amend either the abstract on the online submission form (via Edit Submission) or the abstract in the manuscript so that they are identical.** | The abstract on the online submission has been amended to match the manuscript identically. |
| **7.** **Please include captions for your Supporting Information files at the end of your manuscript, and update any in-text citations to match accordingly**.  Please see our Supporting Information guidelines for more information: <http://journals.plos.org/plosone/s/supporting-information>. | Captions for the Supporting Information files have been included at the end of the manuscript and in-text citations have been updated to match accordingly. |
| **8.** **Please review your reference list to ensure that it is complete and correct.**  If you have cited papers that have been retracted, please include the rationale for doing so in the manuscript text or remove these references and replace them with relevant current references.  Any changes to the reference list should be mentioned in the rebuttal letter that accompanies your revised manuscript.  If you need to cite a retracted article, indicate the article’s retracted status in the References list and also include a citation and full reference for the retraction notice. | Thank you for this. We reviewed the reference list and in-text citations noted some mistakes. These have all been fixed. No papers that have been retracted have been cited. The reference list has been reviewed to ensure it is complete and correct and the following changes have been made:   - Reference number 10 has been updated from the preprint version to the published version. - The link for reference 15 was not working and has been updated with a new webpage and link from the same website. - The link for reference 22 was not working and has been updated with a new webpage and link from the same website. - The article title for reference 42 has been updated. - The article title for reference 50 has been updated. - Reference 64 is new. This has been added for addressing issues of data saturation/ Information Power. - References 64, 67 ,87-97 are new, in response to feedback addressed. |

**Additional Editor Comments**

| **Changes requested** | **Changes made** |
| --- | --- |
| **1. Enhance the Global Relevance**  **Action:** Expand the discussion to address how the findings might be applicable in non-UK contexts, particularly in low- and middle-income countries.  **Specific Examples:** Include a paragraph in the discussion section that explores how the barriers and facilitators identified in your study could manifest differently in healthcare systems outside the UK, especially where digital infrastructure may be less developed. Suggest possible adaptations of your recommendations for these settings. | Thank you for raising this. The following paragraph has been added to the discussion (lines 482-491), which we believe has improved the manuscript.  “Within intervention development, context is critical for the likely success of the intervention [45,86,87]. As such, while the findings of this study are solely derived from participants living in Scotland and England, we are confident that they should be transferable to counties with similar healthcare settings and digital infrastructure, such as other countries in the UK not represented in this sample (i.e., Northern Ireland and Wales) or Australia, Canada, and New Zealand. We are less confident about the transferability of the findings to low-and-middle-income countries, given the lack of public health and accessible sexual health infrastructure in some countries [88,89]. Research is needed to explore the barriers and facilitators to seeking online sexual health information and support and to develop recommendations to overcome the barriers and enhance the facilitators for people from underserved populations in low-and-middle-income countries.” |
| **2. Address Overestimation and Underestimation of Data**  **Action:** Revisit the discussion section to ensure that the recommendations are appropriately scaled to the study’s sample size and qualitative nature. Explicitly state the limitations in generalizing the findings across all underserved populations.  **Specific Examples:** When discussing recommendations like improving digital literacy or providing video/audio options, clarify that these are based on the specific experiences of your participants and may need further validation in other contexts. You could add language such as, “While these recommendations are grounded in the data from our study sample, further research is needed to confirm their applicability across diverse underserved populations.” | Thank you for feedback. We have addressed this in the limitations of the study and have added the following paragraph (lines 551-558):  “the barriers and facilitators and subsequent recommendations presented here are based on the specific experiences of the participants recruited for this study and may not be generalisable across all underserved populations. However, the consistency in our barrier and facilitator findings with previous research with more specific populations suggests that the results of this study and the recommendations generated are transferrable and relevant for a broad range of underserved groups in the National settings mentioned above. Nonetheless, further research is needed to validate the findings in other contexts and confirm their applicability across diverse underserved populations.” |
| **3. Explicitly Acknowledge the Study’s Limitations**  **Action:** Include a dedicated subsection on limitations within the discussion to transparently address the study’s constraints. | Thank you for this feedback. We had previously discussed whether to differentiate or combine the strengths and limitations and it was helpful to have your input here. We have now added two separate sections for strengths and for limitations (lines 508-561) which we believe has provided clarity to the study’s contributions and constraints. |
| **4. Provide a More Nuanced Data Interpretation**  **Action:** Refine the interpretation of your findings to avoid broad generalizations. Highlight the complexity and variability of barriers and facilitators across different subgroups within the underserved populations.  **Specific Examples:** In the results section, when discussing barriers such as privacy concerns, acknowledge that these concerns might vary significantly depending on cultural background, age, and digital literacy levels. Consider adding a sentence like, “Privacy concerns were significant among our sample; however, these concerns may differ in intensity and nature among other subgroups not represented in this study.” | Thank you for raising this. We have expanded each paragraph within the results section to provide high level insights into the subpopulations within our participants who more prominently expressed certain barriers and facilitators, and for whom the recommendations would be most beneficial. We believe this offers a more nuanced interpretation of the data and that this has improved the paper. See the following sections:   - Lines 255-261 - Lines 265-270 - Lines 274-277 - Lines 280-285 - Lines 291-297 - Lines 300-301 - Lines 313-314 - Lines 323-328 - Lines 332-334 - Lines 337-339 - Lines 343-344 - Lines 363-364 - Lines 378-379 - Lines 395-396 |
| **5. Detail Methodological Rigor**  **Action:** Provide more transparency regarding the thematic analysis process. Detail how themes were validated and whether any checks for inter-rater reliability were conducted.  **Specific Examples:** In the methods section, add a brief description of the coding process and whether multiple researchers were involved in analyzing the data. If inter-rater reliability was not assessed, consider acknowledging this as a limitation and suggest that future research could incorporate this step for enhanced rigor.  The manuscript does not mention whether data saturation was achieved. | Thank you for this, we have added the following sentences to the methods and limitations sections (lines provided below) and believe that the changes made have enhanced the clarity on the rigorous methods we used for this study.  We have updated the analysis section to clearly delineate the five steps of Braun and Clarke’s (2006) thematic analysis process that was followed and added a figure (Fig 1) to show these steps with a worked example (lines 165-195).  Within this updated explanation, we have added a sentence to explain the auditing and checking process of the thematic analysis (lines 172-177).  We have also added to the limitations of the paper that, in line with the thematic analysis guide we used (Braun and Clarke, 2006;2021), no inter-rater reliability check was conducted and no formal validation of the themes was conducted (lines 544-551).  We have also added a sentence explaining that our sample size was purposively selected to reach target populations of those from underserved groups and that we sought to attain Information Power, rather than data saturation, in line with Braun and Clarke (lines 109-113). |
| Qualitative research does not aim for generalizability in the same way as quantitative studies, but transferability (the applicability of findings to other contexts) is important. The manuscript does not sufficiently discuss how the findings might transfer to other underserved populations or settings. | Thank you for raising this, along with reviewer two. We have added a paragraph to the discussion to explain the transferability of our findings to other contexts (lines 483-491). In addition, we have updated the limitations to note that, while our findings are consistent with results of previous studies with more specific samples of people from underserved populations, our findings are based on our sample and more research is needed to validate the findings in other populations (lines 552-559). |
| **6. Discuss Policy and Practice Implications**  **Action:** Expand the discussion on the practical implications of your findings, particularly focusing on how your recommendations could be implemented in real-world settings.  **Specific Examples:** For each major recommendation (e.g., improving digital literacy, increasing inclusivity), include a brief discussion of the steps needed for implementation at various levels. For instance, “To implement our recommendation of increasing digital literacy, local health authorities could collaborate with community organizations to deliver targeted training programs that are culturally and linguistically appropriate.” | Thank you for raising this issue. We have provided further detail regarding the need for context-specific implementation beyond those specified in our results and discussion to be considered by service providers and key stakeholders, including the reference to implementation science tools for enhanced rigour in considering practice implications (lines 493-508). |

**Reviewer #1**

| **Changes requested** | **Changes made** |
| --- | --- |
| 1. Which of the qualitative methods has been used? | Thank you for this question. We have detailed the qualitative design (cross-sectional exploratory qualitative approach, line 93) and method (one-to-one participant-led semi-structured interviews, line 155) in the methods section. |
| 2. Add interview questions | Thank you for raising this. Alongside the topic guide with all relevant interview questions for this study as supplementary file (S4 Table), we have now added two example interview questions and explained and provided an example of the follow up prompts to participants responses (lines 136-140) in the procedure section of the methods. |
| 3. Reference should be given for the steps of data reduction | Thank you for highlighting this. We have revised the thematic analysis paragraph and added a figure for further clarity on our data analysis process (lines 155-195). We believe this has been extremely beneficial and improved the manuscript. |
| 4. The criteria used to validate the findings should be added. | Thank you for noting this. In line with the thematic analysis guide we used (Braun and Clarke, 2006, 2021), no formal validation of themes was conducted. We have added this as a limitation to the study (lines 544-546 and 547-549). |
| 5. In a table, the process of abstraction should be mentioned (for a one them from the interview text  You can also use the following Qualitative articles   - [Taghipour, A., Karimi, F., Latifnejad Roudsari, R., Mazlom, S. Coping Strategies of Women Following the Diagnosis of Infertility in Their Spouses: A Qualitative Study. Evidence Based Care, 2020; 10(1): 15-24. doi: 10.22038/ebcj.2020.42136.2120](https://ebcj.mums.ac.ir/article_15306.html) - [Taghipour A, Karimi FZ, Roudsari RL. Exploring iranian women’s perceptions and experiences of their spouses’ behavior towards male factor infertility: A qualitative study. Current Women's Health Reviews. 2020 Feb 1;16(1):60-8.](https://www.researchgate.net/profile/Robab-Latifnejad-Roudsari/publication/337763433_Exploring_Iranian_Women%27s_Perceptions_and_Experiences_of_Their_Spouses%27_Behavior_towards_Male_Factor_Infertility_A_Qualitative_Study/links/5e3995d9458515072d800584/Exploring-Iranian-Womens-Perceptions-and-Experiences-of-Their-Spouses-Behavior-towards-Male-Factor-Infertility-A-Qualitative-Study.pdf) | Thank you for raising this. We were unfamiliar with the term ‘process of abstraction’. Thank you for offering the articles as an example of the Table. We have added a figure for the data analysis process (line 195) which we believe has provided clarity to the process of abstraction and the data analysis steps more broadly. |

**Reviewer #2:**

Overall this is a well written manuscript with sound qualitative methodology. The authors aim to identify barriers and facilitators for seeking online sexual health information and support, and use their findings from semi-structured interviews to make recommendations to support underserved populations. I do feel that the findings from this study add to the medical literature to advance access to online sexual health services for vulnerable or marginalized populations.

| **Changes requested** | **Changes made** |
| --- | --- |
| The main limitation is with the participants-- while diverse in age, ethnicity and education, such wide diversity may limit applicability of the findings to specific populations (ie young adults).  Moreover, certain demographic factors were not included, such as urban/rural location, distance to medical services or establishment with a primary care physician.  I would encourage the authors to acknowledge these limitations in the discussion section. | Thank you for raising this issue. We gave serious consideration to how best to deal with this and have added a paragraph to the limitations of the paper to give context to the diversity of the sample (lines 532-541) and another that discusses the limitations of the diversity of the sample (lines 550-560). We believe this provides important clarity on the sample and who the findings of the study are representative of and applicable to. |
| The authors state 51% lived in "most deprived areas of the UK". How is this defined-- average income? percentage falling below poverty level? | Thank you for this feedback. We have included how we defined this in the results section (line 218):  “51% (n=18) lived in the most deprived areas of the UK (as defined by the Index of Multiple Deprivation [70])” |
